# PlaIMoS: A Remote Mobile Healthcare Platform to Monitor Cardiovascular and Respiratory Variables

**DOI:** 10.3390/s17010176

**Published:** 2017-01-19

**Authors:** Ramses Miramontes, Raúl Aquino, Arturo Flores, Guillermo Rodríguez, Rafael Anguiano, Arturo Ríos, Arthur Edwards

**Affiliations:** 1 Department of Innovation and Technological Development, Rasoft S.A. de C.V., 111 Canario Street, C.P. 28017 Colima, Col., Mexico; miphos@hotmail.es (A.F.); guillermoarb@gmail.com (G.R.); rafaelanguianof@gmail.com (R.A.); arturorogr@gmail.com (A.R.); 2 College of Telematics, University of Colima, 333 University Avenue, C.P. 28045 Colima, Col., Mexico; aquinor@ucol.mx (R.A.); arted@ucol.mx (A.E.)

**Keywords:** health devices, technological platform, e-health

## Abstract

The number of elderly and chronically ill patients has grown significantly over the past few decades as life expectancy has increased worldwide, leading to increased demands on the health care system and significantly taxing traditional health care practices. Consequently, there is an urgent need to use technology to innovate and more constantly and intensely monitor, report and analyze critical patient physiological parameters beyond conventional clinical settings in a more efficient and cost effective manner. This paper presents a technological platform called PlaIMoS which consists of wearable sensors, a fixed measurement station, a network infrastructure that employs IEEE 802.15.4 and IEEE 802.11 to transmit data with security mechanisms, a server to analyze all information collected and apps for iOS, Android and Windows 10 mobile operating systems to provide real-time measurements. The developed architecture, designed primarily to record and report electrocardiogram and heart rate data, also monitors parameters associated with chronic respiratory illnesses, including patient blood oxygen saturation and respiration rate, body temperature, fall detection and galvanic resistance.

## 1. Introduction

### 1.1. Research Background and Motivation

The high incidence of chronic diseases among the general population and an increasing number of elderly, chronically-ill patients is placing ever-increasing demands on healthcare professionals, leading to the development of programs and technological proposals to support innovative at-home health care. Two of the most common chronic diseases that require monitoring as a diagnostic or control tool are cardiovascular and lung diseases.

According to the World Health Organization (WHO), 60% of all worldwide deaths in 2005 resulted from chronic illnesses and half of the 35 million persons dying that year were under the age of 70, with stroke, diabetes, heart disease, cancer and chronic respiratory diseases representing the most common causes [1].

Cardiovascular disease (CVD) was the leading cause of death of approximately 17.5 million persons in 2012, representing approximately 31% of all worldwide deaths from chronic illnesses. Of these deaths, close to 7.4 million were the result of coronary heart disease and approximately 6.7 million were caused by strokes [2]. More recent research carried in 2014 by the British Heart Foundation found that diseases affecting the heart and circulatory system (or CVD) represent the second most common cause of death in the United Kingdom, with a total of around 155,000 deaths (CVD caused 27% of all deaths in the UK) [3]. Heart disease is often caused by an insufficient flow of blood to the cardiovascular muscle, which results in insufficient oxygenation of the heart. Constant monitoring of the heart’s electrical activity is necessary to detect the presence of cardiac arrhythmia to confirm whether the heart has signs of potentially suffering or having suffered a previous heart attack [4].

Electrocardiographic techniques, however, are passive in nature and can only be administered in clinical settings. Their major deficiency is that arrhythmias are fleeting in nature, often occurring sporadically for brief moments over prolonged periods of time. Consequently, many arrhythmias do not present themselves during traditional in-office electrocardiograms (ECGs). The Holter Device, also known as ambulatory electrocardiography, was developed to record patient data for more prolonged periods of two days or so. The Holter Device’s primary disadvantage is that it only records patient information, making it necessary for the patient to return to the medical practitioner to have the data downloaded and analyzed. If a patient has a serious life-threatening episode, the Holter Device cannot help the patient obtain assistance, as it does not transmit information. The PlaIMoS platform presently makes real-time data monitoring, transmission and analysis possible, thus providing a significant improvement in how chronically ill cardiac patients receive medical attention.

Similarly, the World Health Organization estimates around 4 million persons die every year because of chronic respiratory diseases, which represents 6% of all worldwide deaths. The most common chronic lung diseases include asthma, pulmonary hypertension, chronic obstructive pulmonary disease (COPD) and occupational lung diseases, among others. Other factors affecting lung function include chronic bronchitis, cancer, cystic fibrosis, bacterial infections and environmental factors (air pollution, dust, chemicals), which can stress or compromise persons who already have respiratory difficulties.

Although there are no cures for CVDs, various treatments exist to help improve shortness of breath, manage symptoms, and increase the quality of life for people with the disease. 24 h per day patient monitoring of patient respiration rate, blood oxygen saturation, and heart rate provides parameters to determine whether their respiratory and cardiovascular systems are relaxed or stressed [5,6].

Traditional monitoring of vital signs for these chronic illnesses is commonly performed in a hospital bed using specialized medical equipment under the supervision of a doctor, making it difficult to diagnose complications when the patient is mobile [7]. PlaIMoS provides the important advantage of monitoring and transmitting data in real time to the doctor. It also transmits real-time patient body temperature, heart-rate (register the ECG during normal activity), blood oxygen, respiration rate, galvanic resistance and fall data, all while permitting the patient a high degree of mobility. This technology is a step forward as it represents an important diagnostic tool for patients suspected of suffering from chronic health issues or require continuous monitoring to diagnose or confirm a health issues related to cardiovascular disease.

The novelty of the PlaIMoS platform is that integrates health sensors to monitor parameters associated with chronic respiratory illnesses, a wireless sensor network, a 128-bit advanced encryption standard (AES) security level, a patient localization algorithm and software apps for iOS, Android, and Windows mobile devices.

### 1.2. Research Objective

The principal goal of our research was to design, develop and implement a health-monitoring platform to obtain clinical measurements from elderly or chronically ill patients through non-invasive devices to be sent to healthcare providers in real time. The healthcare monitoring system is comprised of a wearable sensor device to constantly monitor heart electrical activity, heart rate, body temperature and fall data, working in conjunction with a fixed measurement station the patient can periodically access to monitor blood oxygen saturation, respiration rate and galvanic resistance. The objectives of this work included developing:

1. A framework to manage and analyze information collected by PlaIMoS platform;
2. A wireless sensor network (WSN) infrastructure that includes a scalable network algorithm;
3. A mobile application to provide real-time monitoring and information access for patients, doctors and caregivers.

## 2. Related Work

### 2.1. Importance of e-Health Platforms

Because of rapid advancements in sensor and communications technologies, governments and the private sector are more intensely incorporating information and communications technology (ICT) in a wide variety of services they offer. The area of health services stands out because of its importance and direct effect on the general welfare of the population. As part of the Millennium Development Goals (MDGs), the World Health Organization (WHO) has called for action to ensure the technological development and universal delivery of health services worldwide by means of various e-Health strategies [8]. Meanwhile, recent advances in the embedded systems, intelligent sensor networks and wireless communication protocol areas enable the integration of large numbers of components combining increased computing capacity, smaller and more long-lasting power sources, expanded wireless communication capability and the miniaturization of sensors and actuators. It is the coordination of these technologies that permit the development of highly specialized applications that offer custom solutions in diverse areas such as environmental monitoring, e-health, smart transportation systems, smart cities, disaster mitigation, etc. One of the main objectives of future technological developments will be to combine mobility with ICT technology to provide a large gamut of utilities to its users, with health and social services being among the most important. To this end, new mobile devices, along with a robust wireless infrastructure that provides seamless connectivity among different fixed, mobile and satellite technologies, will trigger the development of a multitude of e-Health and Tele-Health applications that will revolutionize present-day healthcare models. Presently healthcare models are generally reactive and respond to illnesses after symptoms appear, whereas future healthcare models will be more proactive as their focus will be on prevention and early detection of illness before symptoms surface.

### 2.2. e-Health Platforms

LifeGuard is an important and well-known e-health monitoring system whose principal element is a wearable monitor and the Crew Physiological Observation Device (CPOD). This system has the ability to continuously monitor and report blood pressure, heart and respiration rates, oxygen saturation, and temperature, as well as body position and electrocardiogram data. This system uses Bluetooth to stream the data to a fixed base station. LifeGuard employs a buzzer to alert the user if a sensor records parameters outside the preset limits or if battery power is low [9].

CodeBlue is another hardware and software platform used to monitor and report physiological parameters (blood oxygen, heart rate, electrocardiogram and electromyography) [10]. This platform monitors and transmits patient blood oxygen and heart rate, as well as electrocardiogram and electromyography data. CodeBlue implements a wireless sensor network with a multi-hop routing protocol. This system can locate the patient indoor location using a RF-based localization system. A computer is used to display all information [11]. CodeBlue has a variety of characteristics [12]:

1. Discover and naming: communication pathways between sensors and receiving devices are established;
2. Robust routing: a sensor node reports its data to multiple receiving nodes, thus providing system redundancy;
3. Prioritization of critical data: critical and time sensitive data have priority over other control traffic;
4. Tracking device location: radio frequency (RF) signals are used to track patient and rescue device locations.

Smartvest is comprised of an array of wearable sensors that monitor blood pressure, electrocardiogram (ECG), electromyogram (EMG), electroencephalogram (EEG), temperature, respiration rate, skin response, oxygen saturation and heart rate. The measurements and alarms are sent to a remote monitoring station through a wireless sensor network and all information is displayed at a remote monitoring station [13].

Human++ is a research program whose goal is to develop diverse health applications. A prototype of this program has been created to monitor and visualize electrocardiogram, electroencephalogram and electromyography signals. This prototype uses ultra-wide band to send all information to a computer and personal digital assistant (PDA) device [14].

MEDiSN is a platform that consists of a wireless sensor network with a multi-hop wireless backbone that transmits measurements employing a 128-bit AES encryption scheme to the principal server. This platform monitors heart rate, temperature, blood oxygen, respiration rate, galvanic resistance and electrocardiogram [15].

Signal Processing in a Node Environment (SPINE) is a framework for body sensor network (BSN) applications [16]. The principal structure of this framework employs a coordinator and sensors to monitor patient health [17].

Several methods to detect the users’ physiological state are compatible with the SPINE Project. Gravina and Fortino proposed a basic physiological response using an ECG signal. This project has an algorithm to search for the presence of typical cardiac defense responses inside the generated ECG signal [18]. SPINE2, an evolved version of SPINE, is a framework that offers a task-oriented model to program network sensor nodes. All nodes in the WSN can be dynamically created, discovered and activated by the coordinator on each sensor node [19]. Actually, SPINE2 supports two software sensor platforms: TinyOS2.1 and Texas Instruments Z-Stack. This framework has attained a high independence and programming abstraction level [20].

Researchers at the University of Calabria have developed technologies based on photoplethysmographic (PPG) and ECG signals to estimate patient blood pressure. These technologies help prevent or improve the effects of cardiac diseases caused by blood pressure [21].

The Fall-Mobile Guard project is a real-time fall detection system. This project implements a tri-axial accelerometer to detect falls and determine their severity [22]. PlaIMoS uses identical accelerometer technology and a specific algorithm to detect falls.

The characteristics of general-purpose health platforms are based on studies of architectures and communication technologies for wearable health-monitoring systems [23]. These characteristics include:

1. Health sensors: the types of measurements the platform can perform using a wearable device.
2. Communication system: the communication architecture that transmits data from the wearable device to the collector system.
3. Data security: the security mechanism used to protect the information generated on the platform.
4. Emergency detection: the capacity to launch a push notification if an emergency event occurs.
5. Display devices: the type of devices that display data generated by the platform.

The main features of the various platforms are summarized in Table 1.

## 3. Proposal Architecture

The PlaIMoS platform includes two hardware devices, a WSN infrastructure, and applications (iOS, Android and Windows) to remotely monitor and push alerts.

### 3.1. Architecture

**Figure 1:**
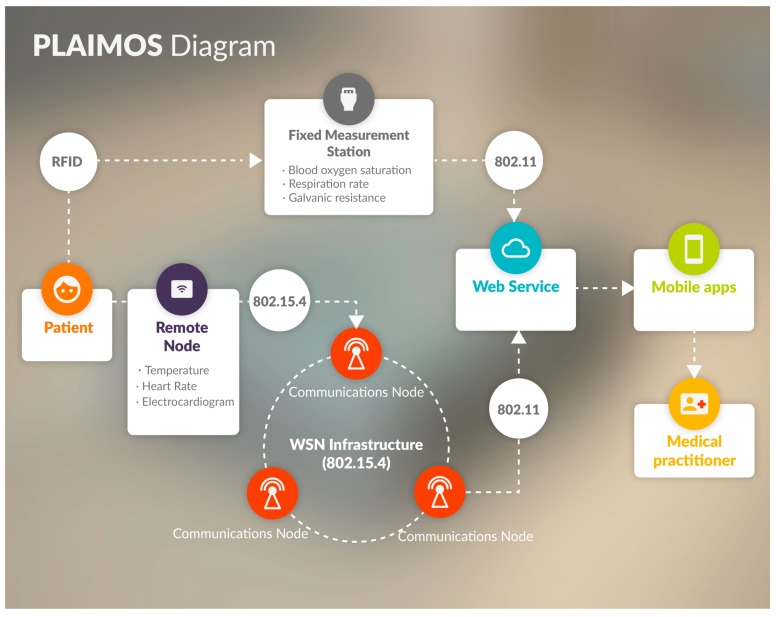
PlaIMoS platform architecture.

PlaIMoS consists of two hardware devices: the PlaIMoS fixed measurement station which measures patient blood oxygen saturation, respiration rate and galvanic resistance, which are identified by radio frequency identification (RFID) sensor and a PlaIMoS remote node that obtains patient body temperature, electrocardiogram, heart rate and fall data. The fixed measurement station is connected to the Internet by means of its Wi-Fi interface which sends all information directly to the PlaIMoS database, located in the cloud. The PlaIMoS remote node employs the IEEE 802.15.4 standard to transmit information to the WSN infrastructure. The Web service receives the information from the WSN infrastructure and sends the data to the PlaIMoS database. All information stored in the database is used by the PlaIMoS API (Web service) and presented in JavaScript object notation (JSON) format for the different apps. The iOS, Android and Windows apps display the information to the participating patients, doctors and caretakers and push alerts when the medical parameters exceed the ranges established by the doctor. This architecture is shown in Figure 1.

### 3.2. PlaIMoS Fixed Measurement Station

**Figure 2:**
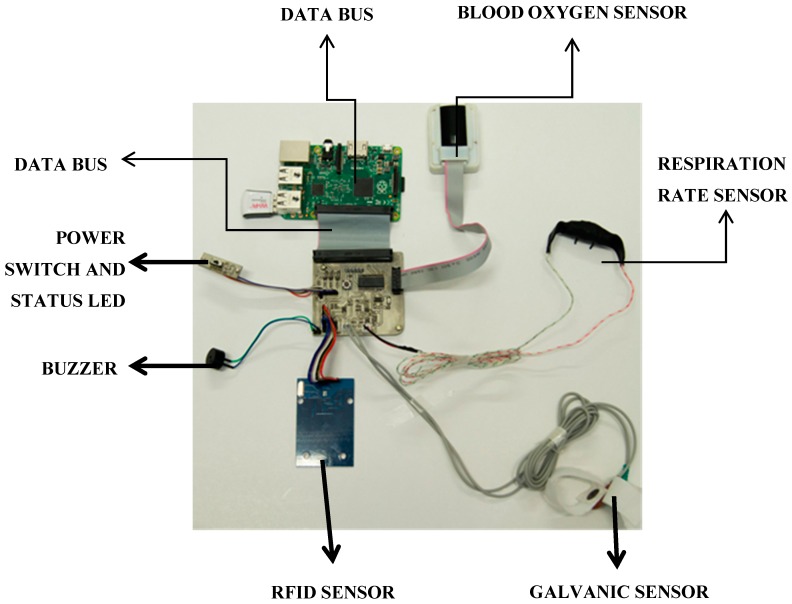
PlaIMoS fixed measurement station hardware.

The PlaIMoS fixed measurement station, designed and manufactured in Colima, Mexico, uses a PIC18F26K22 microcontroller with a 64-KByte flash memory, a 1024-byte EEPROM memory to process information, a MAX30100EFD + optical sensor to monitor blood oxygen, a 40-pin data bus to communicate with a Raspberry Pi card, an airflow sensor to monitor the respiration rate, two electrodes to monitor healthy heart electrical activity, an RFID card reader to identify individual users, a buzzer and an RGB LED to indicate the working state of the device (Figure 2).

The main modules of the fixed measurement station are: general purpose input/output (GPIO), Serial and radio frequency identification (RFID). These modules are part of the patient identification and data collection module. After identifying the patient, the sampled values are automatically assigned to a specific user. The data captured during sampling include blood oxygen, respiration rate, and galvanic resistance.

**Figure 3:**
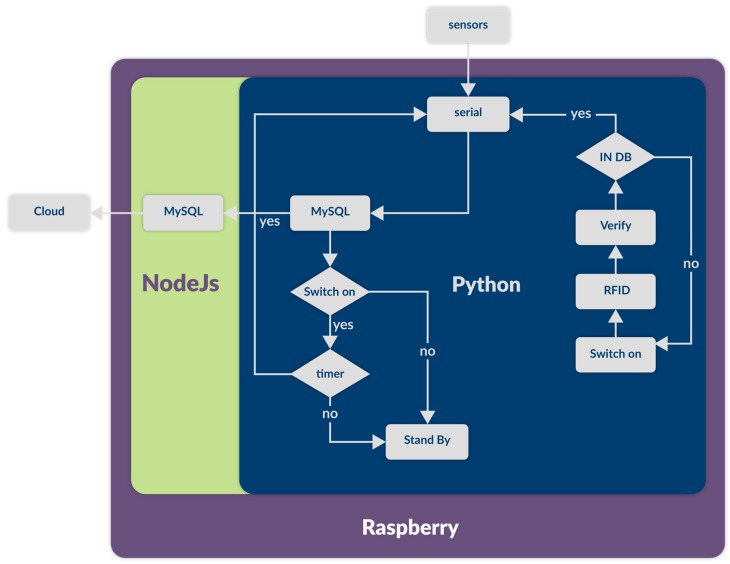
Block diagram of the PlaIMoS fixed measurement station.

The fixed measurement station consists of two principal devices: the sensor unit and a Raspberry Pi chip. The sensor unit receives the sample information from the sensors and transmits the data via a serial link. The Raspberry Pi, programmed in Linux, acts as an intermediary between the sensors and the cloud. Finally, Raspberry Pi stores the sensor readings, user data, and replicates the information in the PlaIMoS database, located in Cloud, as a redundant safety feature. Figure 3 shows a block diagram of the PlaIMoS fixed measurement station with the implemented programming languages, along with all of the processes.

**Figure 4:**
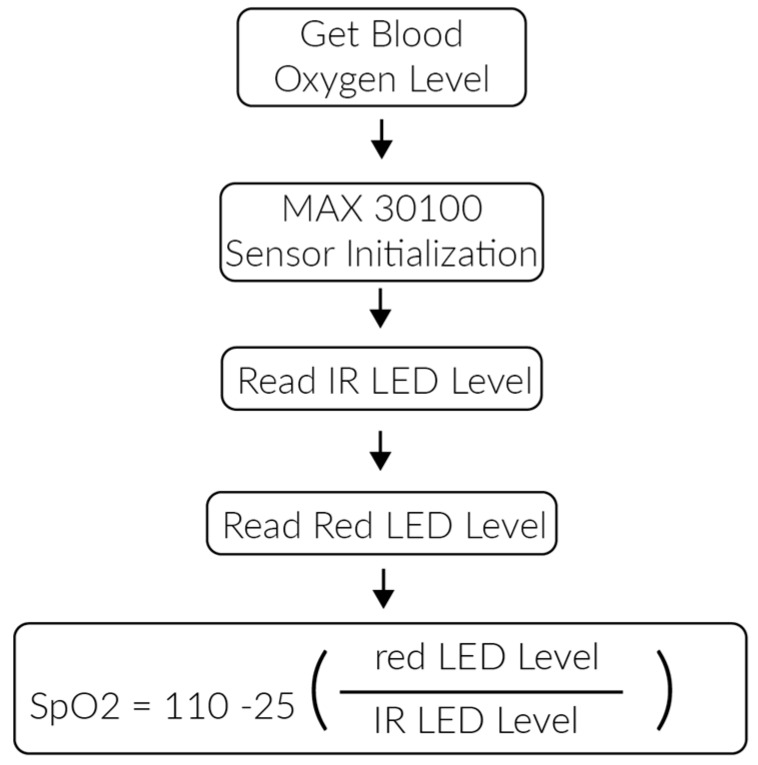
Blood oxygen monitoring algorithm.

The blood oxygen value is obtained using a blood oxygen sensor (MAX30100). This sensor implements two LEDs (infrared and red) that emit light at different wavelengths. The light passes through the user’s finger to be measured by a photodiode. Oxyhemoglobin and deoxyhemoglobin absorbs infrared and red light differently. The different light absorption level is then calculated by the MAX 30100 and applied in the algorithm illustrated in Figure 4.

**Figure 5:**
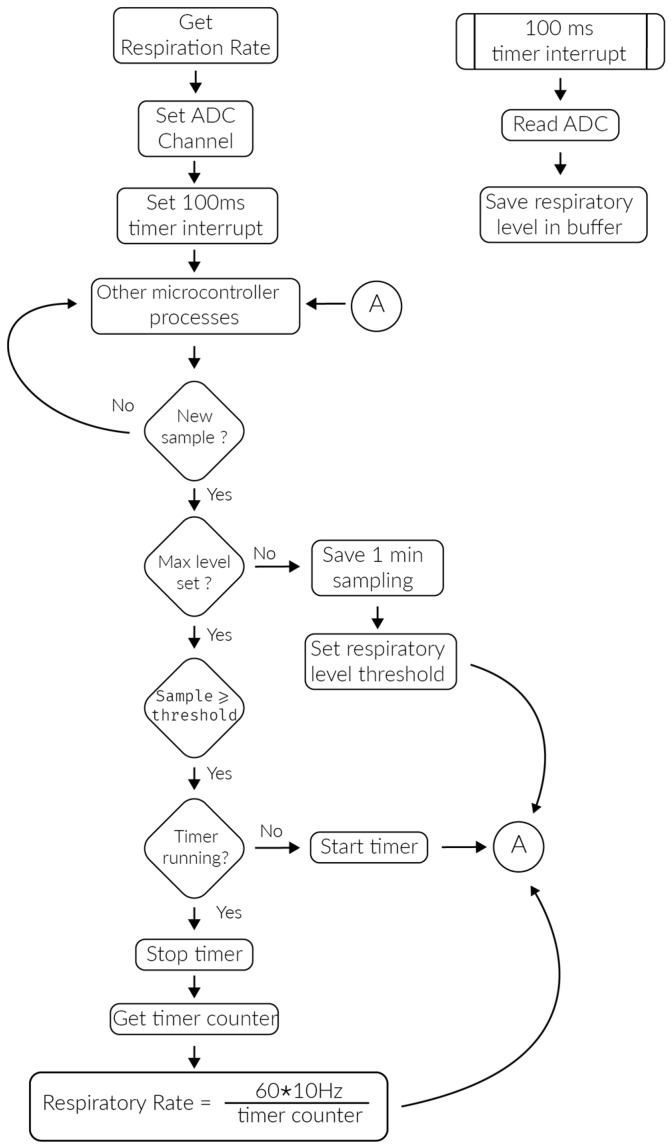
Respiration rate monitoring algorithm.

The respiration rate is calculated measuring the duration of a single breath and extrapolating the average number of breaths per minute. The respiration sensor obtains the threshold of every breath. A timer is then used to measure the time between any two thresholds and to calculate the breaths per minute. Figure 5 represents the algorithm employed to calculate the respiration rate.

**Figure 6:**
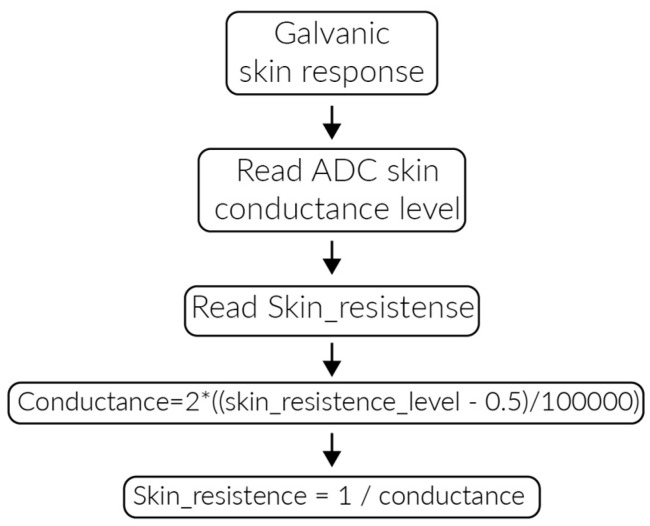
Galvanic skin response monitoring algorithm.

The galvanic skin response monitoring is used to detect the physiological state of users by measuring their sweat gland activity. The galvanic sensor measures the skin response voltage and calculates the value by using an analog to digital converter (ADC) converter. Figure 6 presents the algorithm implemented for this measurement.

**Figure 7:**
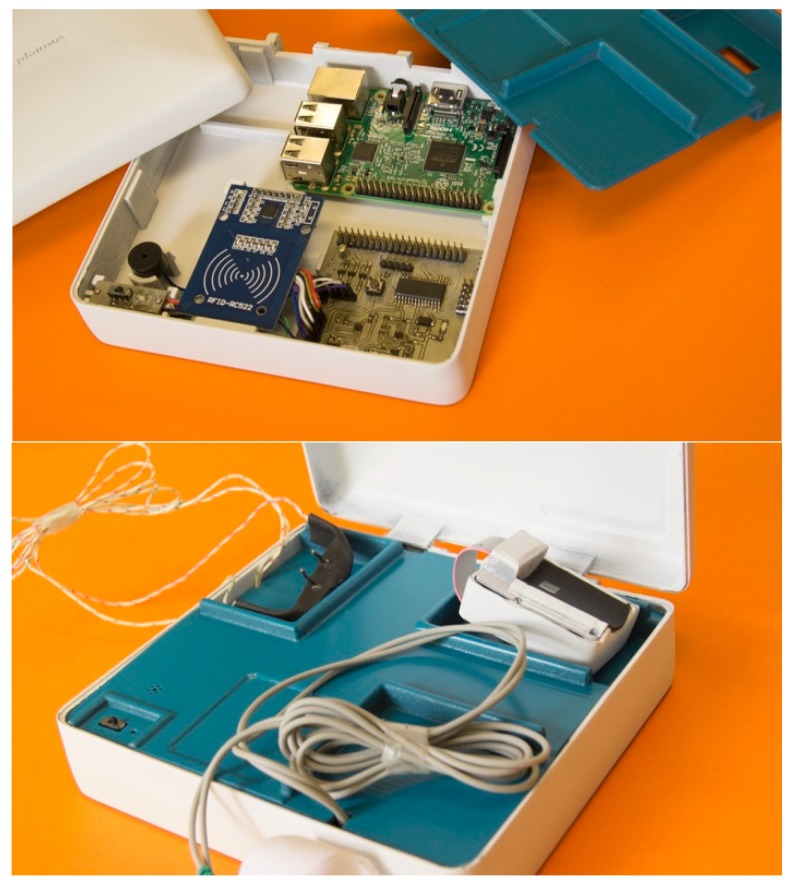
PlaIMoS fixed measurement station opened.

**Figure 8:**
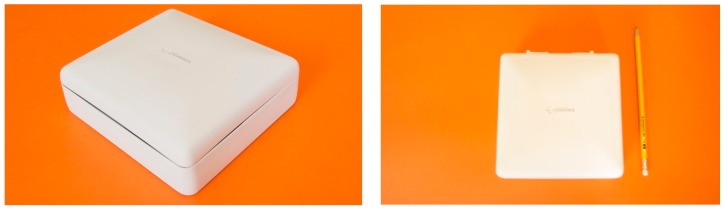
PlaIMoS fixed measurement station closed.

The final version of the PlaIMoS fixed measurement station is shown opened in Figure 7 and closed in Figure 8.

The package information generated by the fixed measurement station sent to the Web service has the following structure (Table 2).

### 3.3. PlaIMoS Remote Node

**Figure 9:**
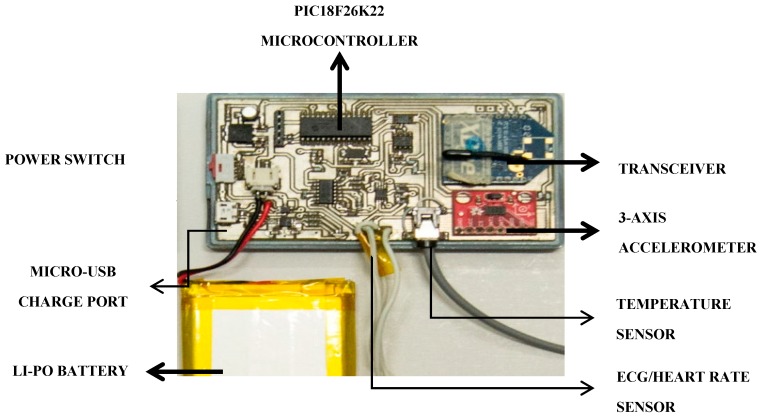
PlaIMoS remote node hardware.

The PlaIMoS remote node, designed and manufactured in Colima, Mexico, uses a 2.4 GHz, 250 Kbps XB24CZ7PIS-004 transceiver, a 64-Kbyte PIC18F26K22 microcontroller, a 1024-byte EEPROM memory chip, a ADXL345 three-axis accelerometer to detect falls, a MCP73833-AMI battery management (4.2 volt at 1 ampere) to charge a Li-Po battery, a Micro-USB port to charge the battery, a slide switch to turn the device on and off, a body temperature sensor, and three electrodes to detect patient heart rate and electrocardiogram data [24] (Figure 9).

The PlaIMoS remote node is a device that patients use to continuously monitor their vital signs such as temperature, fall detection, heart rate and electrocardiogram. It employs a 12h lithium battery and applies IEEE 802.15.4 wireless technology to transfer data in a WSN infrastructure. This device acquires the electrical signals generated by the heart and provides the corresponding waveform as an electrocardiogram.

**Figure 10:**
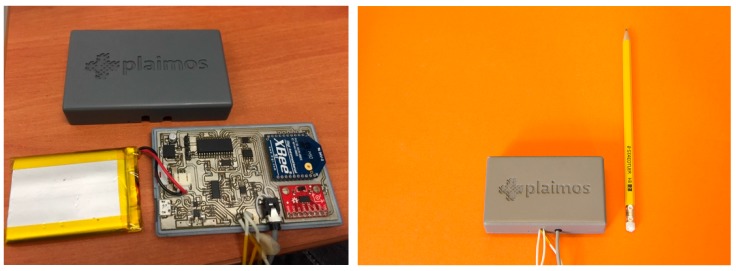
The PlaIMoS remote node device.

This information is then processed for later viewing on mobile devices to acquire the patient heart rate. Additionally, to better monitor patients who are suffering from infections, body temperature, and fall detection are also monitored. The final version of the PlaIMoS remote node is shown in Figure 10.

The received signal is preprocessed by a series of filters and amplifiers to obtain the signal in a range of between 0 and 3.3 V, which is processed analogically and converted to a digital signal by a 10-bit converter. The same analog to digital converter (ADC) is also used to process the electronic signal from a resistive temperature detector (RTD). Using a Wheatstone bridge and a dedicated algorithm [24], it is possible to accurately obtain the patient’s body temperature. A digital accelerometer (MXL345) measures acceleration experienced by the user along three axes to determine the user’s physical position. Finally, a dedicated algorithm [25] is used determine whether the patient is in a vertical or horizontal position. If a possible uncontrolled transition from the vertical to horizontal position occurs, a push package is transmitted alerting persons monitoring the user of a possible fall.

**Figure 11:**
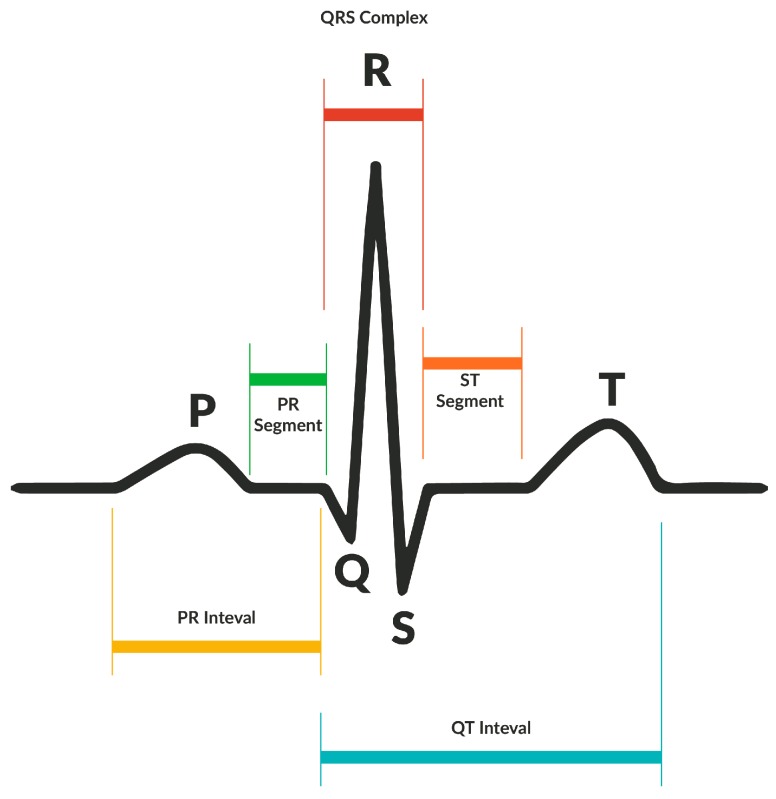
QRS Complex.

Heart rate detection is generated by electrical signals generated by each of the heart’s ventricles. The constriction and relaxation of the ventricles as they pump blood through the body generates an electrical wave, which is known as the QRS complex (Figure 11).

**Figure 12:**
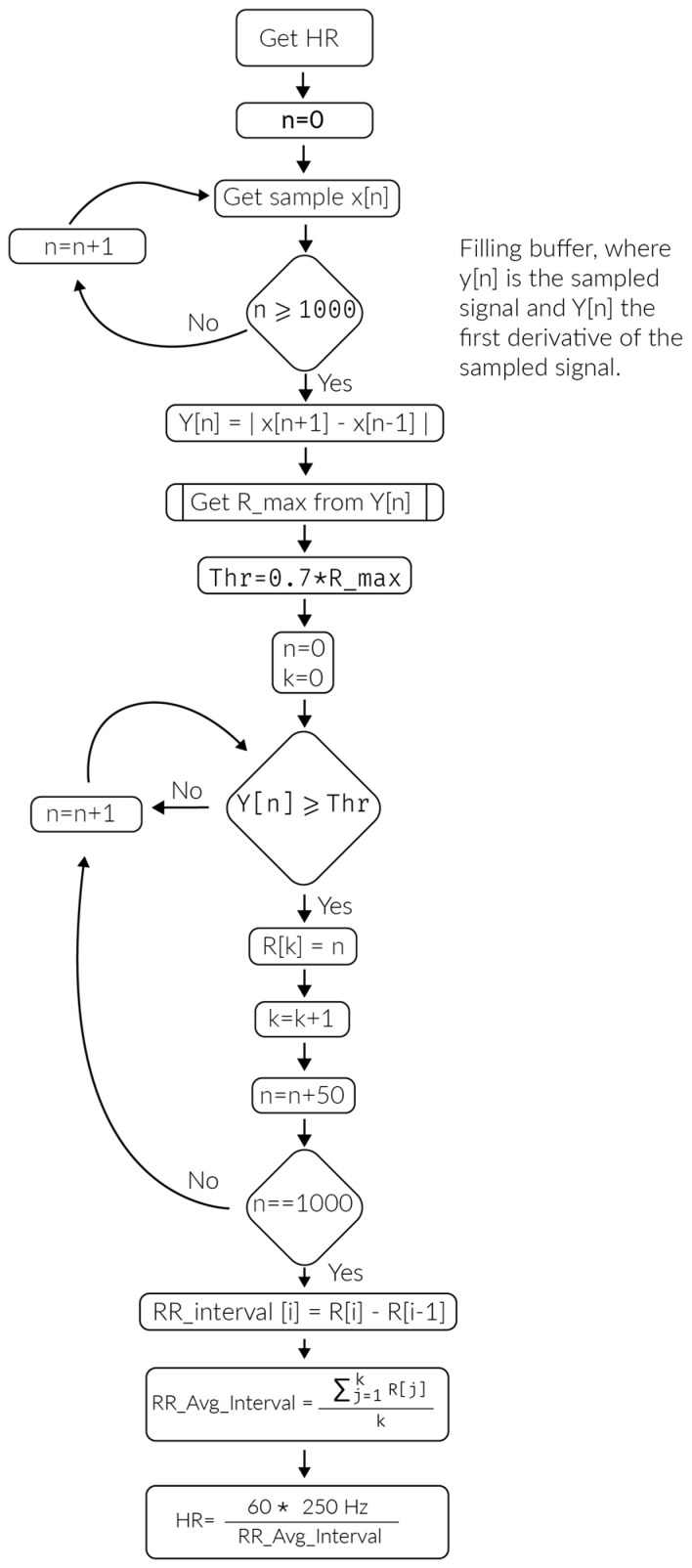
Heart rate detection algorithm.

To obtain the patient’s heart rate, it is necessary to know how many QRS complexes are generated per minute. Several methods are described below [26]. The device uses a sampling frequency of Fs = 250 Hz so that each sample is taken at a time interval of Ts = 1/Fs, which is important because it does not need to sample the heart rate for a full minute to produce reliable data. Instead, it only records the interval between two QRS complexes at random and its algorithm extrapolates the beats per minute. However, in order to obtain greater accuracy and eliminate probable reading disturbances, the device compiles a total of 1000 samples to calculate the heart rate, based on an average of RR (ridge peak to ridge peak) intervals (Figure 12).

**Figure 13:**
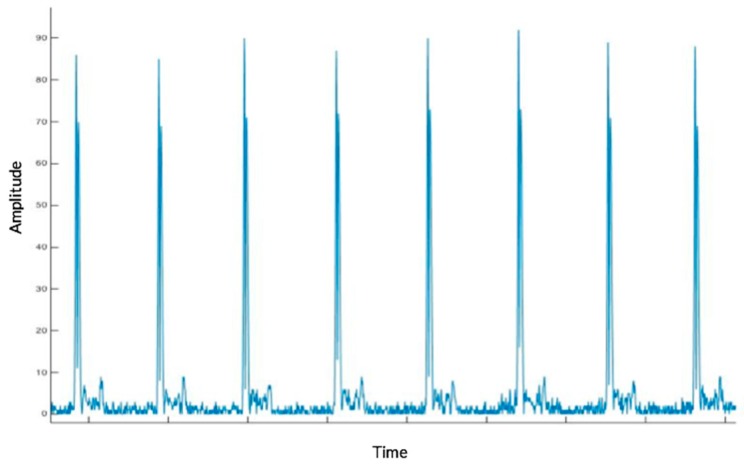
Electrocardiogram First Derivation.

When a thousand samples are stored (n), the absolute value of the first derivative of the samples is obtained in order to provide a precise measurement of the R ridge as shown in Figure 13. Employing this process provides PR and ST intervals that are considerably lower compared to the peaks of the QRS complex and negative values disappear because of the algorithm used. Additionally, the subsequent 50 samples are ignored, thus avoiding capturing the same QRS complex twice or possibly disrupting an ST interval. Each time a QRS complex is identified, it is saved to determine the average time interval between two QRS complexes.

**Figure 14:**
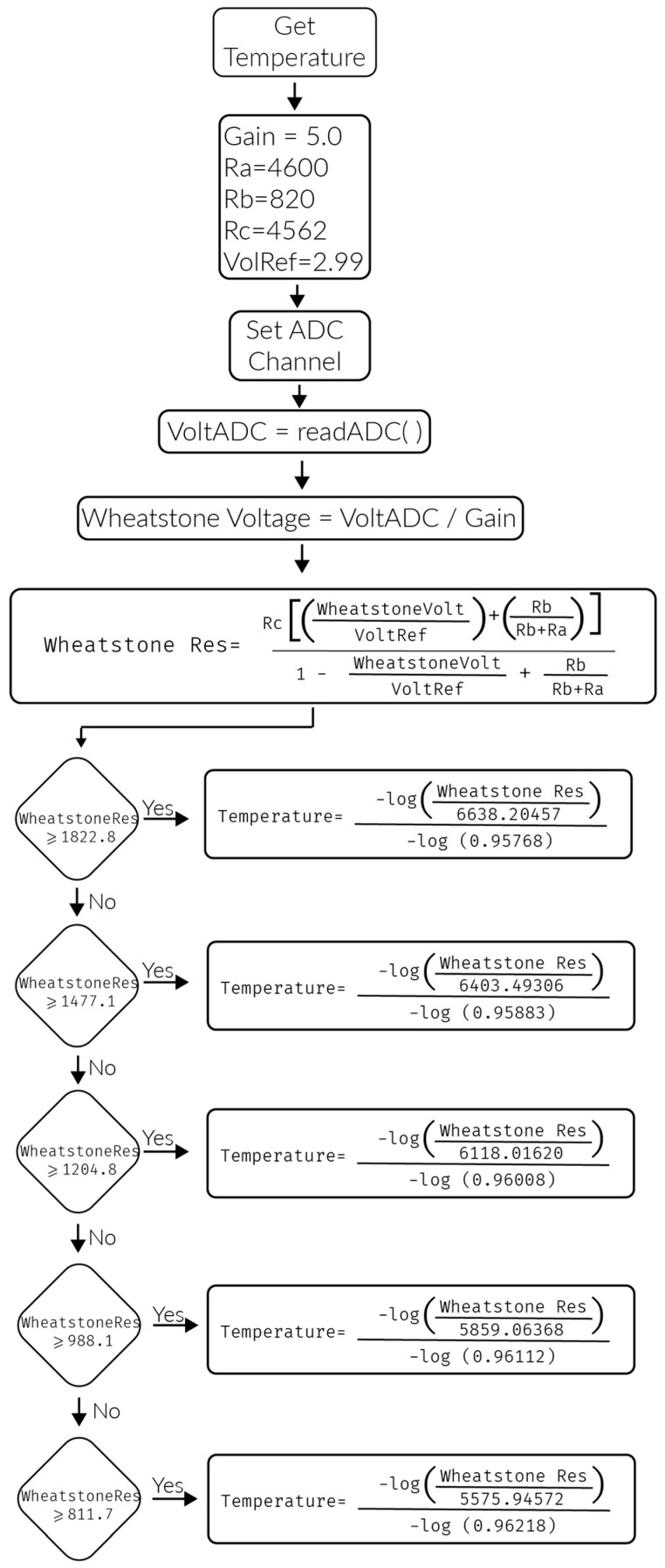
Temperature monitoring algorithm.

Subsequently, the average interval is calculated for the 1000 samples and then applied in the following equation:

(1) $$HR=\;\frac{60*250\;\mathrm{Hz}}{RR\_Avg\_Interval}$$

where 250 Hz corresponds to the sampling rate, 60 refers to the number of seconds per minute and RR_Avg_Interval is the average time between the intervals formed by the QRS complex in the sampled signal. Patient body temperature is obtained by means of a resistance temperature detector and subsequently applying a monitoring algorithm proposed by the manufacturer [24] as shown in Figure 14.

**Figure 15:**
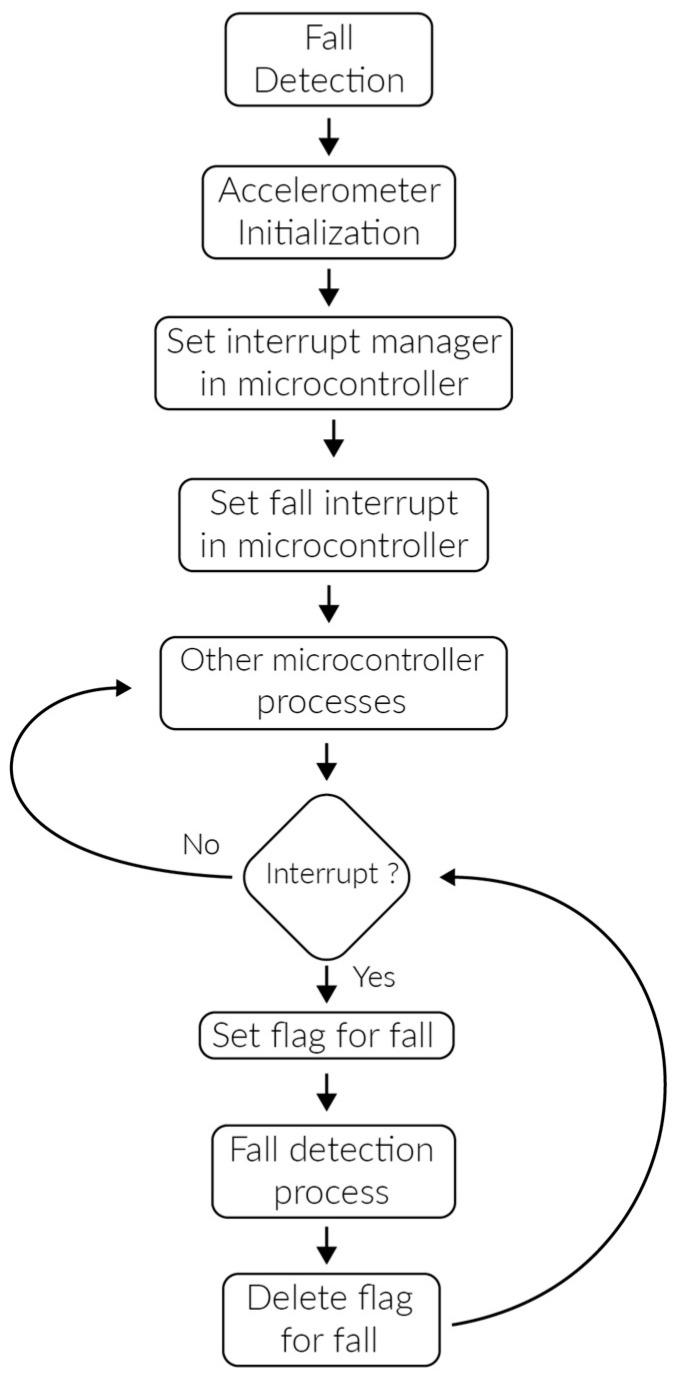
Fall detection process.

Fall detection is detected by means of an ADXL345 three-axis accelerometer, which includes an algorithm to detect real falls and forward a notification to the microcontroller when a fall occurs. The fall detection process is presented in Figure 15.

### 3.4. Wireless Sensor Network Infrastructure

The WSN Infrastructure is a network composed of low power communication devices: a PlaIMoS communication node and a PlaIMoS remote node. The aim of the WSN Infrastructure is to generate a coverage area that can monitor physical variables by forming a wireless sensor network.

The PlaIMoS communication node is responsible for receiving and managing the information received through the PlaIMoS remote node using IEEE 802.15.4 and forwarding the information to a Web service using IEEE 802.11. This device can perform gateway, cluster or sink functions.

CSMA/CA is a multiple access method which employs carrier sensing. In CSMA/CA, nodes listen to the shared medium to determine whether other nodes are transmitting to avoid packet collisions which may occur if more than one node attempts to transmit on the same channel at the same time. When a channel becomes available, nodes transmit their complete packet data, thus insuring the transfer of information [27]. This method was implemented in the WSN:

- It is scalable; because it is hierarchical, the networks created under this protocol have the ability to incorporate other devices without losing their efficiency. This feature is achieved in large part because each network device possesses a specific role (PlaIMoS remote node, cluster, gateway and sink);
- It provides accurate user location; because of its distributed scheme and its centroid localization algorithm, the PlaIMoS remote node can be located with an accuracy that depends on the number of nodes in the WSN infrastructure;
- It permits user mobility; PlaIMoS-Routing combines a routing protocol developed to operate in static networks where the components are motionless, along with a scheme in which the network nodes can be in constant motion;
- It employs redundant links; during network formation, PlaIMoS-Routing stores each node’s link in a routing table, thus ensuring alternate routes if the data cannot be transmitted via the main link. PlaIMoS-Routing determines the main links through the creation of a metric (determined as weight), which is formed by the intensity of the received signal (RSSI), the number of hops and the lifetime of each node (TTL);
- It is fault tolerant; because of possible redundant links, PlaIMoS-Routing allows the network to continue operating should any link or node fail. If a node has the ability to re-establish its operation, the protocol allows it to rejoin the network and adopt the best suited role at that moment;
- It implements security schemes; PlaIMoS-Routing encrypts data using AES 128, which authorizes the persons or devices authorized to access and use the information generated in the network;
- It utilizes a reactive/ proactive scheme; PlaIMoS-Routing can operate in two different schemes, meaning it can be programmed to operate exclusively to detect any device possessing information that needs to be transmitted, thereby reducing the energy resources required by the network devices, or it can be scheduled to continuously transmit information.

The PlaIMoS remote node sends continuous sampling or electrocardiogram packets to the WSN infrastructure. Continuous sampling packets contain a packet identifier, a patient identifier, a temperature value, a heart rate value, a physical position value (falls) and the patient’s location (Table 3).

Electrocardiogram packets possess a packet identifier, a patient identifier and 80 sample values that comprise the electrocardiogram wave (Table 4).

All information that is received by the WSN Infrastructure is forwarded by the PlaIMoS communication node in gateway mode to the PlaIMoS communication node in sink mode, where it is forwarded to a Web service in the cloud. This service receives the information and stores it in the PlaIMoS database, which is responsible for obtaining information from the database to make it accessible to the apps in a standardized JSON format. The PlaIMoS-Routing communications protocol is formed by four types of communication nodes:

1. Remote node; the device that patients use to continuously send their vital signs to the WSN using IEEE 802.15.4;
2. Cluster device; the device responsible for group conformation within the network which manages and receives information from remote sensing node using IEEE 802.15.4;
3. Gateway device; the device that acts as the communication link between cluster devices to forward information using IEEE 802.15.4;
4. Sink device; the device that receives and forwards all information from WSN infrastructure to the PlaIMoS server using IEEE 802.15.4, 802.11 and 802.3.

**Figure 16:**
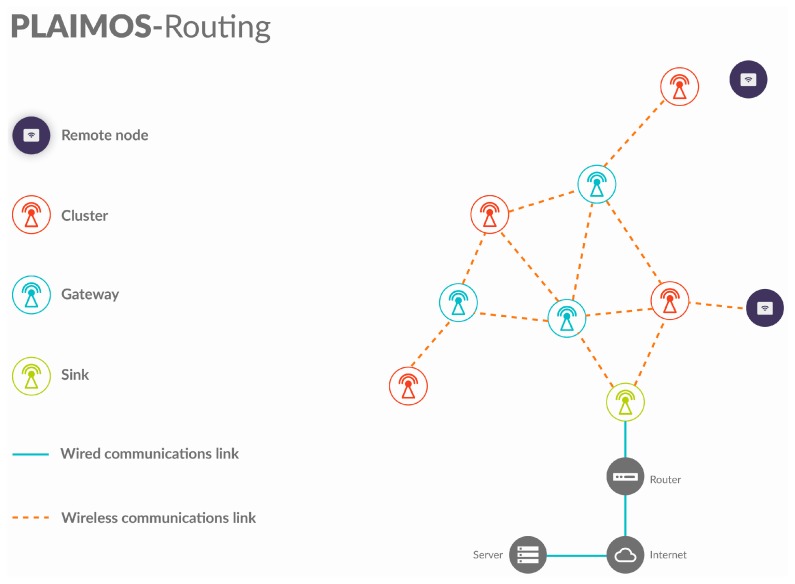
PlaIMoS-Routing.

Figure 16 shows a diagram of a conventional network using the PlaIMoS-Routing communications protocol.

### 3.5. Software Application

The mobile application for the PlaIMoS platforms allows the healthcare provider to view patient body temperature, heart rate and electrocardiogram values, as well as review the latest measurements pertaining to respiration rate, blood oxygen and real-time galvanic resistance.

This application was developed for iOS (iPhone), Android and Windows Phone in order to cover most of the market users. For safety reasons, persons authorized to use the PlaIMoS platform must register a username and password to access PlaIMoS App via smartphone.

When healthcare providers begin their sessions in PlaIMoS, they first view the list of assigned patients and select the specific patient to consult details. Once the healthcare provider selects the patient that he/she wants to query, he/she can scroll down a list that contains the patient’s most recent measurements. The caregiver can easily scroll a list that efficiently provides the following information:

- Name of the measurement performed
- Date and time of the measurement performed
- Value of the measurement performed
- Minimum measurement value
- Maximum measurement value

Healthcare providers can change the minimum and maximum values of each patient measurement to personalize the acceptable range for each person. If any measurement exceeds the range limits, a push notification is executed in real time to notify the person responsible for monitoring the patient [28]. The push notification runs as follows:

1. The Web service detects if a measurement is outside the normal preset range.
2. The Web service solicits the notification server (Apple, Google and Microsoft Server) to send a push notification to a particular device.
3. The notification server receives the request and forwards the notification push to the indicated device.
4. The device (smartphone) emits a visual and audible alert with the incidence reported by the Web service regardless of whether the device is locked or the application is running.

The PlaIMoS App allows the doctor to view the electrocardiogram of patients in real time. Once entering the electrocardiogram view, the healthcare provider can fully analyze the graphs generated because he/she can detain the real-time data stream to zoom in and analyze a specific waveform.

## 4. Results

The PlaIMoS pilot study was carried out over a 1h period at the University Regional Hospital with the objectives of:

- relaying information through the WSN Infrastructure using the PlaIMoS-Routing protocol
- locating the indoor location of PlaIMoS remote nodes
- transmitting clinical parameters through the PlaIMoS fixed measurement station
- generating alarms when patient clinical parameters exceeded the present ranges (temperature and patient falls)
- viewing clinical patient information

### 4.1. Pilot Study

**Figure 17:**
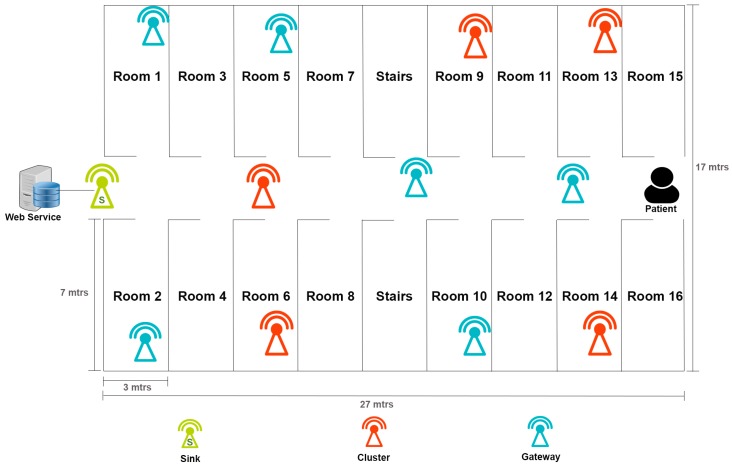
First floor of the hospital (pilot study scenario).

Twelve PlaIMoS communication nodes were used to test the PlaIMoS platform (five in Cluster mode, six in Gateway mode and one in Sink mode). One PlaIMoS remote node was also used to form a distributed network in the University Regional Hospital. The 12 PlaIMoS communication nodes were distributed on the first floor of the hospital; of these, eight were installed in rooms and four in the main hallway. The test area measured 459 m^2^ (27 m × 17 m) and consisted of 16 patient rooms and two stairwells (Figure 17).

The PlaIMoS remote node was used by a patient for the pilot study which included the transmission of temperature, heart rate, electrocardiogram and fall detection data. The PlaIMoS fixed measurement station was placed in one of the rooms (Room 1) to be used by the patient. This device was connected to the Internet via IEEE 802.11 to send measurements to the Web service.

In order to validate our concept, actual hospital equipment was used to compare measurements obtained by PlaIMoS platform. The equipment utilized included:

1. Criticare nGenuity [29] (to obtain electrocardiogram, heart rate, blood oxygen and respiration rate data)
2. Microlife MT3001 [30] (to determine body temperature)

Participants in the pilot study included a PlaIMoS engineer, a patient and a doctor.

### 4.2. Results of the WSN Infrastructure Using the PlaIMoS-Routing Protocol

The PlaIMoS-Routing protocol was installed in the 12 PlaIMoS communication nodes of this study. For 60 min, the patient used the PlaIMoS remote node and moved among the 16 hospital rooms, pausing for 3 min in each. During this period, the PlaIMoS remote node generated 6650 packets, of which 6429 were received correctly by the PlaIMoS communication node that served as the sink. Only 221 packets failed to be received, indicating a packet loss of 3.32%.

**Figure 18:**
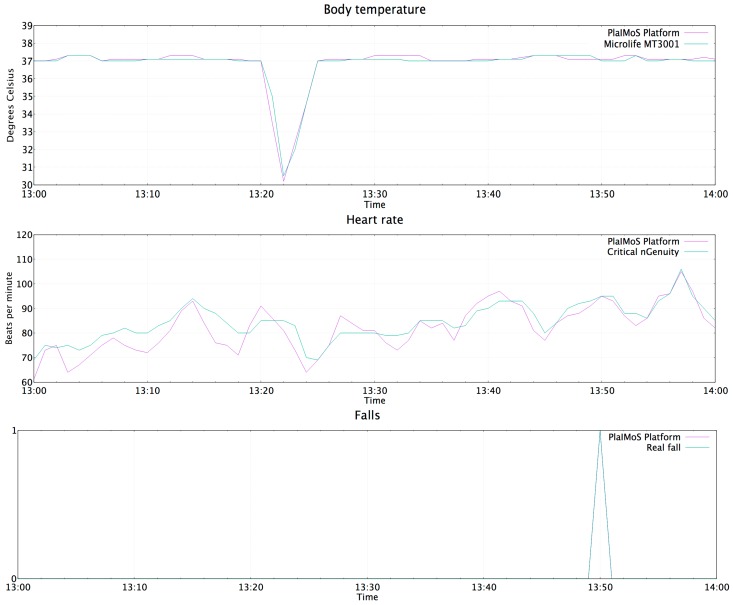
Values obtained by the PlaIMoS remote node.

Figure 18 shows user temperature, heart rate and fall detection data obtained from the PlaIMoS platform and professional hospital equipment which was used to validate readings when relevant.

### 4.3. Results of Indoor Location for PlaIMoS Remote Node

**Figure 19:**
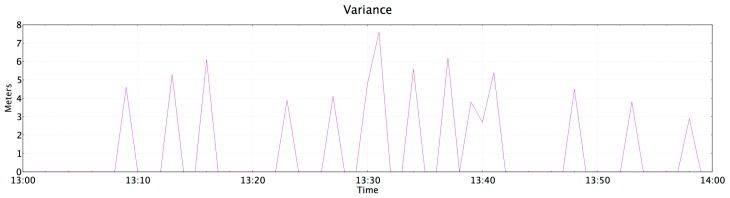
Variance in meters between real location and the location saved.

Results show that the centroid localization algorithm used by PlaIMoS was able to locate the remote mobile node to within 7.6 m. To obtain this result, the data packages transmitted were reviewed in real-time while the patient moved among the rooms. Figure 19 shows the variance in meters between real location of the patient and the location saved it by PlaIMoS.

The accuracy depends on the number of nodes in the WSN infrastructure. The location accuracy increases proportionally with the number of network nodes.

### 4.4. Results of the PlaIMoS Fixed Measurement Station

The PlaIMoS fixed measurement station was used by the patient for 3 min because it corresponds to the time the test patient was in the room 1 of the University Regional Hospital. During this period the station sent 180 packages to the Web service, which translates into 180 records in the PlaIMoS database. In total, 180 packets were successfully received, resulting in a packet loss rate of 0%. It is important to note that the blood oxygen saturation and respiration rate readings were identical between the PlaIMoS platform and the actual hospital equipment. Importantly, the University Regional Hospital did not possess equipment to validate patient galvanic skin response.

**Figure 20:**
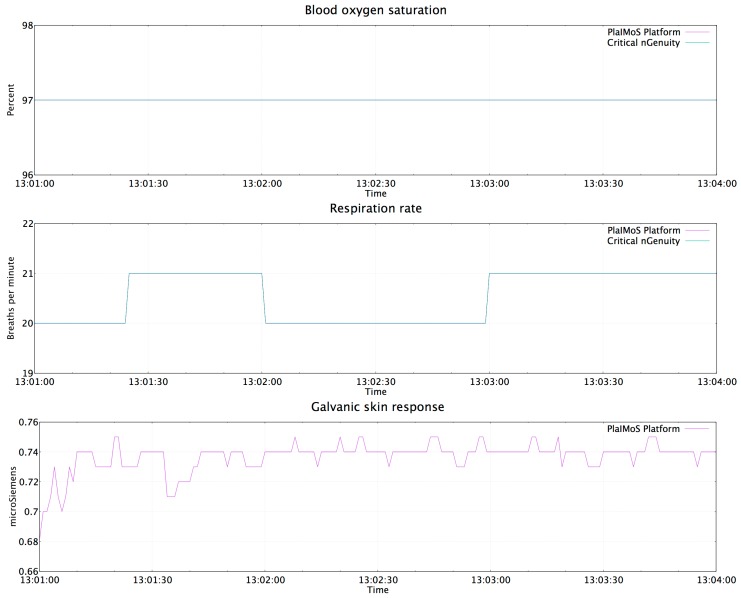
Values obtained by the PlaIMoS fixed measurement station.

Figure 20 presents the data obtained for patient blood oxygen saturation, respiration rate and galvanic skin response by the PlaIMoS Platform.

### 4.5. Push Notification in Case of Abnormalities

The PlaIMoS platform has an alarm system (push notification) that is executed at the time of an anomaly. For the system to detect that an abnormal event has occurred, the healthcare provider must set the minimum and maximum values of the physiological variable to be monitored. If the measurement received from any of the sensors is outside the range, a push notification will be transmitted to the healthcare provider’s smartphone. A total of two alarms were executed (on queue) during the study in order to validate push notification functionality:

1. Temperature of 30 °C. (a cold object was placed over the temperature sensor)
2. The patient let himself fall to represent a faint

Results show that the smartphone received the temperature alarm 7 s after the PlaIMoS remote node sent a measurement to the Web service and the fall alarm was received 6 s after the simulated incident. The time difference is due, in large part, to the time it takes the Apple, Google and Microsoft servers to receive the requests from the PlaIMoS platform and for them to forward the notification to the smartphone.

### 4.6. App to Display Patient Information

**Figure 21:**
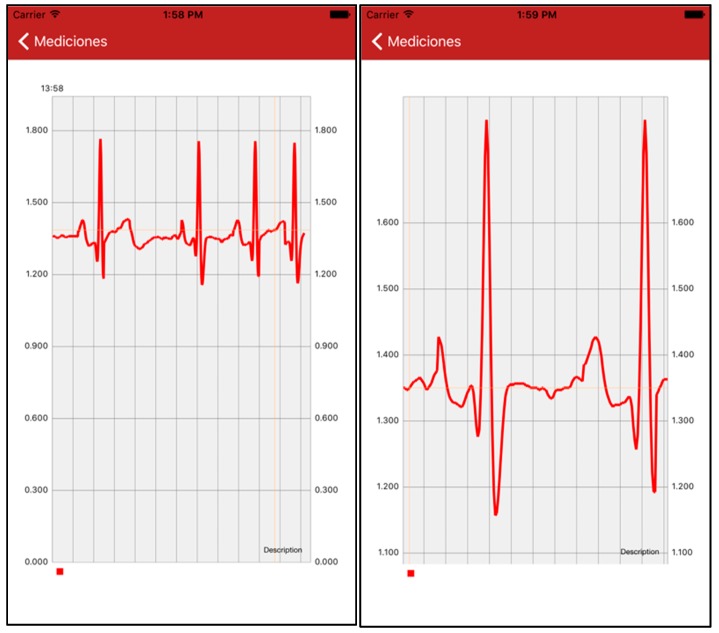
Electrocardiogram View.

During the hospital test, a registered physician was responsible for constantly checking the patient's temperature, blood oxygen, heart rate, respiration rate, galvanic resistance and location, using the PlaIMoS App. Furthermore, electrocardiogram information was automatically updated every second, where a still shot was produced and magnified for more detailed study (Figure 21). During the hospital test, the PlaIMoS App correctly presented all required information without crashing because the application was developed natively for each operating system [31].

## 5. Conclusions and Future Work

Our proposal represents an important step and a major challenge in terms of usability and acceptance by users. Mobile remote technologies used as part of health monitoring and maintenance strategies will grow in importance in the future. The PlaIMoS platform performed well in a hospital setting, securely monitoring and reporting patient information in real-time circumstances. As PlaIMoS can also be used as an at-home cost effective alternative or expansion of traditional clinical healthcare services, it can function alone or potentially in conjunction with health information systems (SIS), hospital information systems (HIS) and electronic medical records, through integration and management of health databases.

PlaIMoS platform was validated to currently monitor and report six parameters (temperature, electrocardiogram, heart rate, blood oxygen, respiration rate and fall data), as well as the indoor location of the patient. Our next is to increase the number of parameters PlaIMoS can handle (i.e., adding blood pressure, blood sugar etc.) and develop software to cross reference these parameters and provide a more in-depth diagnostic tool for medical practitioners. Future work will also include validation of the galvanic skin response recorded by the PlaIMoS platform using hospital equipment.

## Figures and Tables

**Table 1:** e-health platform characteristics.

Platforms/Characteristics	LifeGuard [9]	Code Blue [10]	Smartvest [13]	Human++ [14]	MEDiSN [15]	SPINE2 [19]	PlaIMoS
Health sensors	Blood pressure ECG heart rate respiration rate oxygen saturation temperature body position	Blood oxygen heart rate ECG EMG	Blood pressure ECG EMG EEG temperature respiration rate skin response oxygen saturation heart rate	ECG EEG EMG	ECG heart rate blood oxygen	ECG heart rate temperature respiration rate skin response fall detection	ECG heart rate temperature blood oxygen respiration rate skin response fall detection
Communication system	Bluetooth	Wireless sensor network	Wireless sensor network	Ultra-wide band communication	Wireless sensor network	Wireless body sensor network	Wireless sensor network
Data security	Not provided	Not provided	Not provided	Not provided	128-bit AES encryption into the WSN	ZigBee security layer	128-bit AES encryption into the WSN and database
Patient localization	Not provided	RF-based localization system for indoor location	GPS	Not provided	Not provided	Not provide	centroid localization algorithm for indoor location
Emergency detection	Buzzer is used to alert the user	Not provide	Automatic alarms at the remote monitoring	Not provided	Alarm through back-end server	Alarm through wireless body sensor network	Push notification from server to display device
Display devices	Base station computer	Computer	Remote monitoring station	Computer PDA	Not provided	Computer	Computer tablets smartphones

ECG = electrocardiogram; EMG = electromyogram; EEG = electroencephalogram; AES = advanced encryption standard; PDA = personal digital assistant; GPS = global positioning system.

**Table 2:** Fixed measurement station package.

Package_id	Patient_id	Blood_oxygen	Respiration_rate	Galvanic_resistance
Int	Int	Float	Smallint	Float

**Table 3:** Continuous sampling package.

Package_id	Patient_id	Temperature	Heart_rate	Fall	Location
Int	Int	Int	Float	Int	Int

**Table 4:** Electrocardiogram package.

Package_id	Patient_id	Sample 1	…	Sample 80
Int	Int	Float	…	Float
